# Human Papillomavirus Infections in Cervical Samples From HIV-Positive Women: Evaluation of the Presence of the Nonavalent HPV Genotypes and Genetic Diversity

**DOI:** 10.3389/fmicb.2020.603657

**Published:** 2020-11-25

**Authors:** Catia Sias, Valerio Guarrasi, Claudia Minosse, Daniele Lapa, Franca Del Nonno, Maria Rosaria Capobianchi, Anna Rosa Garbuglia, Paola Del Porto, Paola Paci

**Affiliations:** ^1^Laboratory of Virology, Lazzaro Spallanzani National Institute for Infectious Diseases, IRCCS, Rome, Italy; ^2^Dipartimento di Ingegneria Informatica, Automatica e Gestionale “A. Ruberti”, Sapienza Università di Roma, Rome, Italy; ^3^Laboratory of Pathology, Lazzaro Spallanzani National Institute for Infectious Diseases, IRCCS, Rome, Italy; ^4^Department of Biology and Biotechnology “C. Darwin”, Sapienza University, Rome, Italy

**Keywords:** Human papillomavirus, HPV mixed infection, genetic variability, intraepithelial lesions, HPV vaccine

## Abstract

Non-nonavalent vaccine (9v) Human papillomavirus (HPV) types have been shown to have high prevalence among HIV-positive women. Here, 1444 cervical samples were tested for HPV DNA positivity. Co-infections of the 9v HPV types with other HPV types were evaluated. The HPV81 *L1* and *L2* genes were used to investigate the genetic variability of antigenic epitopes. HPV-positive samples were genotyped using the HPVCLART2 assay. The L1 and L2 protein sequences were analyzed using a self-optimized prediction method to predict their secondary structure. Co-occurrence probabilities of the 9v HPV types were calculated. Non9v types represented 49% of the HPV infections; 31.2% of the non9v HPV types were among the low-grade squamous intraepithelial lesion samples, and 27.3% among the high-grade squamous intraepithelial lesion samples, and several genotypes were low risk. The co-occurrence of 9v HPV types with the other genotypes was not correlated with the filogenetic distance. HPV81 showed an amino-acid substitution within the BC loop (N75Q) and the FGb loop (T315N). In the L2 protein, all of the mutations were located outside antigenic sites. The weak cross-protection of the 9v types suggests the relevance of a sustainable and effective screening program, which should be implemented by HPV DNA testing that does not include only high-risk types.

## Introduction

Cervical cancer is the fourth most frequent cancer in women (Ferlay et al., 2019). Statistical data suggest that almost 99% of cervical cancers are related to Human papillomavirus (HPV) infections (Walboomers et al., 1999; De Martel et al., 2012). For 70% of the women who become infected during the first half of their lives, only 10% of these infections become persistent, and a minority of these progresses to cervical cancer (Woodman et al., 2007). The evolution toward intraepithelial lesions is related to HPV genotypes. The International Agency for Research on Cancer has indicated the 15 α-types as high-risk (HR) genotypes: HPV16, 18, 31, 33, 35, 39, 45, 51, 56, 58, 59, 66, 68, 73, and 82 (Muñoz et al., 2003). These genotypes are responsible of more than 90% of all cervical cancers (Luostarinen et al., 2018), and they are also involved in anal and oropharynx cancers (Garbuglia et al., 2020). While the low-risk (LR) types are predominantly involved in the development of genital warts, only HPV6 and HPV11 are responsible for recurrent respiratory papillomatosis. This disease is very difficult to treat, and it often has fatal outcomes (Garbuglia et al., 2020).

In developed countries, the cervical cancer screening program started 40 years ago, and it has contributed to reduced incidence of and mortality from cervical cancers (Peirson et al., 2013). However, the prevalence of invasive cervical cancer (ICC) remains high in Africa, southeast Asia, and eastern Europe, where preventive medicine is not practiced or is ineffective. Indeed, in these geographical areas, the population screening program reaches between 6% and 8% of the female population (Sudenga et al., 2013). Therefore, a prophylactic vaccine represents a powerful tool for eradication of cervical cancer.

In 2006, two L1 virus-like particle (VLP) vaccines were licensed: Cervarix, a bivalent vaccine that contains VLPs of the HPV16 and HPV18 HR types, and Gardasil, a quadrivalent vaccine that includes VLPs of the HPV16 and HPV18 HR types, plus the two: HPV6 and HPV11 LR types. These vaccines prevent 70% of cervical cancers, and the tetravalent vaccine (4v) can also prevent epidermodysplasia associated to HPV6 and HPV11 infections (Garland et al., 2007). In December 2014, a non-avalent vaccine (9v) was licensed by the United States Food and Drug Administration, and in June 2015, this was also approved by the European Commission (Lopalco, 2016). VLPs of five additional HR types, HPV31, 33, 45, 52, and 58, were included in the 9v. Theoretically, the 9v can prevent up to 93% of cervical cancers (Brotherton et al., 2017), with 100% seroconversion documented, for antibody titers that remain stable over decades (Nygård et al., 2015). Countries that have included high vaccination coverage have obtained 73% to 85% reductions in infections by the 9v HPV types, and 41% to 75% decreases in high-grade lesions [cervical intraepithelial neoplasia (CIN) grade 2 or worse] for up to 10 years after vaccination (Drolet et al., 2019). In addition, clinical trials carried out after the vaccine licensing have demonstrated an efficacy of up to 100% against vulvar and vaginal intraepithelial neoplasia for the bivalent and quadrivalent vaccines administrated to virus-naïve women. Despite these encouraging results, data have appeared in the literature that describe high-grade squamous intraepithelial lesions (HSIL) or cervical tumors linked to HPV types that are not included in the current vaccines. Amaro-Filho et al. (2020) identified HPV73 in cervical cancer, which was previously considered a LR type. HPV73 and HPV56 have also been frequently associated with vaginal intraepithelial neoplasia 2/3, and HPV51 and HPV59 with vulvar intraepithelial neoplasia 2/3, in Japan (Sugase and Matsukura, 1997). In a study carried out among women with HSIL or ICC, 4.5% of the patients with CIN 2/3, and 3.9% of those with cervical cancer were positive to one or multiple LR HPV types. HPV54 and HPV73 have been observed in cervical cancers, with HPV42, 54, 62, 67, 70, and 82 reported for CIN2 samples (Siegler et al., 2019). In Korea, HPV56, 68, and 73 were detected in adenocarcinoma, and HPV59 in squamous cell carcinoma (Park et al., 2019), while HPV82 was reported as the most frequently observed HPV type in HSIL in Oman (Al-Lawati et al., 2020). Although the 9v has shown broad efficacy against the five additional HR types (Toft et al., 2014), it is not known whether it can cross-protect against all non-vaccine HPV types. The lifespan of the cross-neutralizing antibodies following HPV immunization is also not known (Toh et al., 2019). In particular, among HIV positives, the cross-neutralizing antibody titers were lower than those of HIV-negative people (Weinberg et al., 2012).

These aspects are particularly relevant among HIV-infected women, who have a high rate of HPV infections and harbor multiple HPV infections more persistently, in comparison to HIV-negative women (Palefsky et al., 1999; Garbuglia et al., 2012). In a 4-year follow-up study on HIV-positive women who were vaccinated with the 4v, incidence and persistence of HPV51 and HPV39 types were observed. This suggests that previous data on the 4v cross-neutralization activities described in HIV-negative women did not extend to HIV-positive women (Toh et al., 2019).

The aim of the present study was to determine the prevalence of HPV types included and not included in 9v in low-grade squamous intraepithelial lesions (LSIL) and HSIL among HIV-positive women. Based on these observations, a further aim was to determine the probability that the 9v genotypes co-exist with the other HPV types that are circulating among HIV-positive women in Italy. Furthermore, the L1 and L2 genes were analyzed for single infections (SIs) and multiple infections (MIs), to investigate the genetic variability of the antigenic epitopes in these two groups (i.e., SIs, MIs).

## Materials and Methods

### Study Population

This was a cross-sectional study that included HIV-positive women who had been screened for HPV-related diseases at the gynecology Outpatient Service of the “L. Spallanzani” National Institute for Infectious Diseases (IRCCS, Rome). The Institutional Ethical Committee approved the study protocol (n. 42/13). All of the methods were carried out according to the ethical standards of the Institutional and National Research Committee and the Declaration of Helsinki.

Cervical cell samples were obtained using a cytobrush, and were preserved in 2 mL phosphate-buffered saline [24], and stored in ice until they were delivered to the Laboratory of Virology, where they were processed. Cervical smears were prepared for cytological examination (Pap-tests) concomitant with sampling for HPV testing. Cytological diagnosis was formulated according to the 2001 Bethesda system (Solomon et al., 2002), and classified as normal or abnormal (diskaryosis) findings. This latter group included atypical squamous cells of undetermined significance, LSIL, or HSIL. All SIL cases were confirmed by colposcopies and biopsies.

### HPV-DNA Analysis

Approximately 1.8 mL cervical swabs samples were centrifuged at 1,500 × *g* for 5 min. The pellet was resuspended in 1.5 mL phosphate-buffered saline, and 20 μL proteinase K (Qiagen, Hilden, Germany) was added, with the samples then incubated at 56°C for 10 min. An aliquot of 800 μL was used to extract the nucleic acids (Qiasymphony automatic instrument; Qiagen, Hilden, Germany), according to the manufacturer instructions. The residual samples were immediately frozen and stored at−80°C until used for further investigations.

All of the samples were tested for amplifiable DNA using β-globin primers (Saiki et al., 1985; Sias et al., 2019), and HPV was detected using the MY09/11 primers, as previously described (Garbuglia et al., 2012). The PCR products were visualized by 1.8% agarose gel electrophoresis with ethidium bromide staining. HPV-positive samples were genotyped (HPVCLART2 assay; Genomica, Madrid, Spain; Garbuglia et al., 2012). This assay is based on nucleic acid amplification technology followed by visualization in low-density microarrays of the HPV PCR product. This system detected 35 HPV types: 15 HR types (HPV16, 18, 31, 33, 35, 39, 45, 52, 56, 58, 59, 66, 68a and 68b, 73, and 82), 15 LR types (HPV6, 11, 41, 42, 43, 44, 54, 62, 71, 72, 81, 83, 84, 85, and 89), and four potential carcinogenic types (HPV26, 51, 53, and 70). Samples, that could not be typed by Genomica were analyzed by Sanger sequencing, as described in Sias et al. (2013). Briefly, the MY09/11 PCR products were purified with Qiaquick for PCR products (Qiagen, Hilden, Germany) and directly sequenced using a genetic analyzer (AB3130; Applied Biosystems, Life Technology, Forster, CA, United States). The HPV types were identified and quantified by comparison of the DNA sequences with those available in GenBank, through the BLAST server (BLAST, 2020), and they were classified according to De Villiers (De Villiers, 2013).

### L1 and L2 Sequences

To study the L1 genetic variability of HPV81, we selected a subset of HPV81 SIs (*n* = 6) and a subset of HPV81 MIs (*n* = 4). HPV81 was chosen because it has been frequently observed in association with LSIL and HSIL grade lesions (Minosse et al., 2010; Annunziata et al., 2018; Ge et al., 2019), though it is considered a LR type, and little information is available on its genetic variability. The PCR to amplify the *L1* region was based on two PCRs that were combined to obtain the full length of the *L1* sequences. All of the primer mixes were directed against highly conserved motifs of the HPV81 genome. *L1* region mix A generated fragments of approximately 850 bp in length, while the mix B gave a PCR product of 950 bp (Table 1). Each mix consisted of 50 μL 1x TaqGold buffer (TermoFisher Scientific, United States), 1.5 mM MgCl_2_, 0.2 mM each dNTP, 0.5 μM each primer, 1.5 U TaqGold polymerase (TermoFisher Scientific, United States), and 10 μL DNA extracted from cervical samples. The assay conditions were: 15 min at 94°C, and 35 cycles: 94°C 40 s, 58°C for 45 s, and 72°C for 115 s, followed by a final extension at 72°C for 10 min.

**TABLE 1:** Primers used for the amplification of *L1* and *L2* of HPV81.

Gene	Mix	HPV81-L1/L2 primer	Location (nt)*	Sequence (5′→3′)	Amplicon length (bp)
L1	1 round A	OuterSense1	5647–5663	ACCACCGTTCCTTTGTC	930
		Antisense1	6577–6550	CTCAGCAGCCATTTGTAAATAATCTGGA	
	2 round A	InnerSense1	5724–5745	AGCCCCTTCTATAGTCCCTTCG	853
		Antisense1	6577–6550	CTCAGCAGCCATTTGTAAATAATCTGGA	
	1 round B	OuterSense2	6525–6544	ATATTTGCAATACCACCTGT	954
		Antisense2	7478–7458	CAATACACATATAAATACAAC	
	2 round B	InnerSense2	6550–6577	TCCAGATTATTTACAAATGGCTGCTGAG	928
		Antisense2	7478–7458	CAATACACATATAAATACAAC	
L2	1 round	OuterSense	4390–4410	CTGCACATTGTTTTGTACTAT	1524
		Antisense	5914–5895	AGACACAGGTGTGGGAGGCA	
	2 round	InnerSense	4437–4457	TACATTGTATACTCTATTGTA	1477
		Antisense	5914–5895	AGACACAGGTGTGGGAGGCA	

*Nucleotide positions refer to the prototype AJ620209.

The L2 gene was amplified by nested PCR amplification using the L2 outer sense and antisense primers (round I), and the L2 inner sense and antisense primers (round II; Table 1). The composition of the reaction mixture was as used for the *L1* gene. After a denaturation step at 94°C for 15 min, amplification reactions were for 35 cycles for each round, and consisted of 94°C for 45 s, 56°C for 45 s, and 72°C for 90 s. Amplification products were purified (QIAquick PCR purification spin kits; Qiagen GmbH, Hilden, Germany) and sequenced directly using Big Dye Terminator Cycle Sequencing Ready Reaction kits, and analyzed using a sequencing system (AB3130; Applied Biosystems, Forster, CA, United States). The PCR and sequencing were performed in duplicate.

All of the sequences were aligned with an HPV81 prototype sequence (GenBank accession number, AJ620209). The sequences of the *L1* and *L2* genes were submitted to the NCBI GenBank database, and were assigned accession numbers (MT547553 – MT547561).

Amino-acid sequences were aligned using CLUSTALW (Thompson et al., 1997). We used a self-optimized prediction method (NPSA, Combet et al., 2000; Prabi, 2020) to estimate the β-turn structure. β-Turns are very frequently the sites of interactions in proteins and peptides, both because they are topologically biased to occur on the surfaces of proteins, and because their structures present the side chains of corner residues, which are optimal for molecular recognition. The Hamming distance (Hamming, 1986; Farci et al., 2000) was calculated using online software (Hacksparrow, 2019). The Pearson distance and phylogenetic tree were calculated using the MEGA10 software (Kumar et al., 2018). The phylogenetic tree of the L1 protein was calculated according to Anisimova (Anisimova and Gascuel, 2006), using online software (Phylogeny, 2020).

### Co-occurrence Probability Determination

To compute the co-occurrence probability between 9v types and the other HPV types detected by Genomica, we evaluated the empirical (or experimental) probability of an event that is an “estimate” that an event will occur based upon how often the event occurred after collecting data from an experiment in a large number of trials (Bickel and Doksum, 1977; Durrett, 1996). This type of probability is based upon direct observations. Each observation in an experiment is called a trial. The empirical probability is given by Eq. (1; Bickel and Doksum, 1977; Durrett, 1996):

(1) $$P\left(E\right)=\frac{\text{number}\text{of}\text{times}\text{the}\text{event}\text{occurs}}{\text{total}\text{number}\text{of}\text{trials}}.$$

In our analysis, the event *E* is the co-occurrence between the reference 9v non-avalent genotypes (called genotype *A*) and one of the others (called genotype *B*), and the total number of trials is the number of times the reference genotype occurs in the given dataset (Bickel and Doksum, 1977; Durrett, 1996). Thus, we called this in our analysis *P(E)*, as the co-occurrence probability of the event *E*. Finally, we defined the complementary probability *C(E)* of event *E* as the probability of not observing *A* given B, as in Eq. (2):

(2) C(E)= 1-P(E).

Thus, the complementary probability is maximal [i.e., *C*(*E*) = 1] when *P* = 0, which means that the presence of A rules out the possibility to have B.

### Least Squares Method

To determine correlations between the co-occurrence and genotype distance (both Hamming and Pearson genetic distances), we used least squares analysis, which is a standard approach in regression analysis (Feller, 1971; Bickel and Doksum, 1977; Kelley, 1994; Bretscher, 1995; Durrett, 1996; Casella and Berger, 2002; Larson and Farber, 2003; Moore and McCabe, 2003). To assess the strength of the correlations, the *R*^2^ index was evaluated. *R*^2^ is a statistical measure that represents the proportion of the variance for a dependent variable that is explained by an independent variable or variables in a regression model. Whereas correlations explain the strength of the relationships between independent and dependent variables, *R*^2^ explains to what extent the variance of one variable explains the variance of the second variable. So, if *R*^2^ of a model is 0.50, then approximately half of the observed variation can be explained by the model inputs. An *R*^2^ of 1 indicates that the regression predictions perfectly fit the data (Feller, 1971; Kelley, 1994; Bretscher, 1995; Casella and Berger, 2002; Larson and Farber, 2003; Moore and McCabe, 2003). The regression linear model was calculated only on the points where the co-occurrence probability was greater than zero. Finally, we computed the 95% confidence intervals of the least squares linear regression line. The 95% confidence interval is the range of values for which you can be 95% certain contains the true mean of the population. As the sample size increases, the range of the interval values narrows, meaning that you know that mean with much more accuracy compared with a smaller sample (see Supplementary Data 1).

All of the computations were completed using open-source tools, with Python version 3.6 (Python, 2020); in particular, and with the following libraries: pandas (Pandas, 2020), numpy (NumPy, 2020), scipy (Scipy, 2020), matplotlib (matplotlib, 2020), and seaborn (Seaborn, 2020).

## Results

### HPV Prevalence and Cytological Findings

**FIGURE 1:**
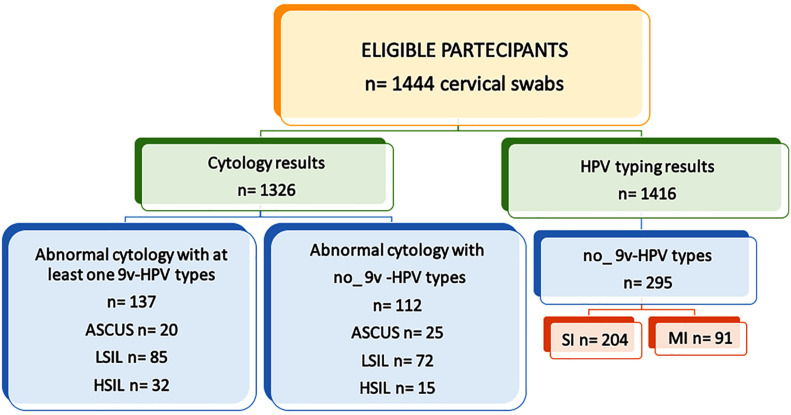
Study design flow chart. Human papillomavirus (HPV) DNA testing results and cytological findings. 9v: nonavalent vaccine; ASCUS, atypical squamous cells of undetermined significance; LSIL, low-grade squamous intraepithelial lesion; and HSIL, high-grade squamous intraepithelial lesion.

Between 2008 and 2012, 1,444 cervical samples from HIV-positive women were screened for HPV DNA. The median age of the women was 52.63 years (range, 27–89 years), and none of them had been vaccinated. A total of 602 samples were HPV DNA positive (41.7%) and 21/602 (3.5%) were β-globin PCR negative. Also, 336/602 (55.8%) were HPV SI, and 266 (44.2%) were HPV MI. For eight samples, the Genomica CLART 2 Array failed to detect any HPV genotypes. These samples were submitted to Sanger sequencing, which allowed the identification of their HPV types: HPV13, HPV55, HPV69, HPV102 (*n* = 2), HPV118, and HPV120 (*n* = 2). Overall, 9v types (HPV6, 11, 16, 18, 31, 33, 45, 52, and 58) were found in 51.0% (307/602) of HPV-positive samples: 39.3% (132/336) were SI and 65.8% (175/266) were MI. No 9v types represented 49.0% (295/602) of HPV infections, 69.2% (204/295) of which were SI, and 30.9% (91/295) were MI (Figure 1).

**FIGURE 2:**
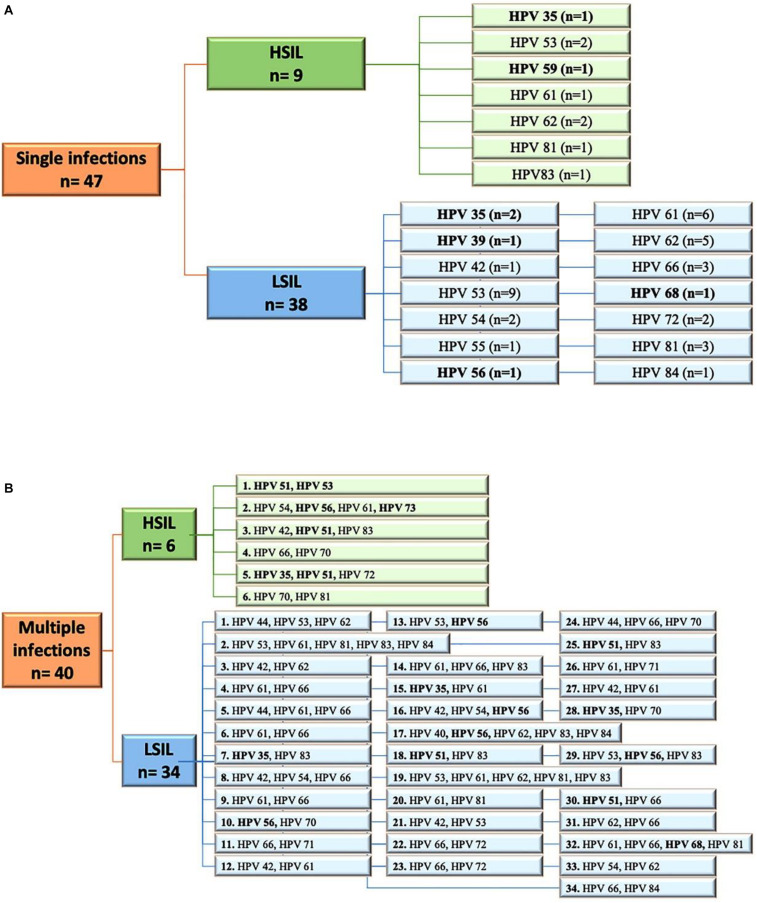
**(A)** Flow diagram of HPV single infections in low-grade squamous intraepithelial lesion (LSIL) and high-grade squamous intraepithelial lesion (HSIL) pap smears. **(B)** Flow diagram of HPV multiple infections observed in LSIL and HSIL pap-smears. HR, high risk HPV types; LR, low risk HPV types. All HR are highlighted in bold. N, number.

The women were also screened for cervical lesions. Cytological findings were available for 1326 patients. A total of 286 patients (21.6%) had cervical dysplasia of any grade (i.e., LSIL or HSIL). No intraepithelial cervical cancer was seen. Here, 97 patients showed abnormalities for atypical squamous cells of undetermined significance or atypical glandular cells of undetermined significance, whereas 911 patients had normal cytological results. LSIL was observed in 231 specimens, HSIL in 55 specimens. Among the LSIL findings, 74 samples (32.2%) harbored HPV types not included in 9v. Fifteen HSIL were non-9v HPV types (27.3%); nine were SI, and six were MI (Figure 2). Considering SI and intraepithelial lesions, LR HPV42, 54, 72, and 84 were detected only in LSIL, HPV83 only in HSIL, and HPV61, 62, 81 in both (Figure 2A). Five MI, which harbored only LR HPV types, were linked to LSIL (Figure 2B).

### HPV Co-occurrence

**FIGURE 3:**
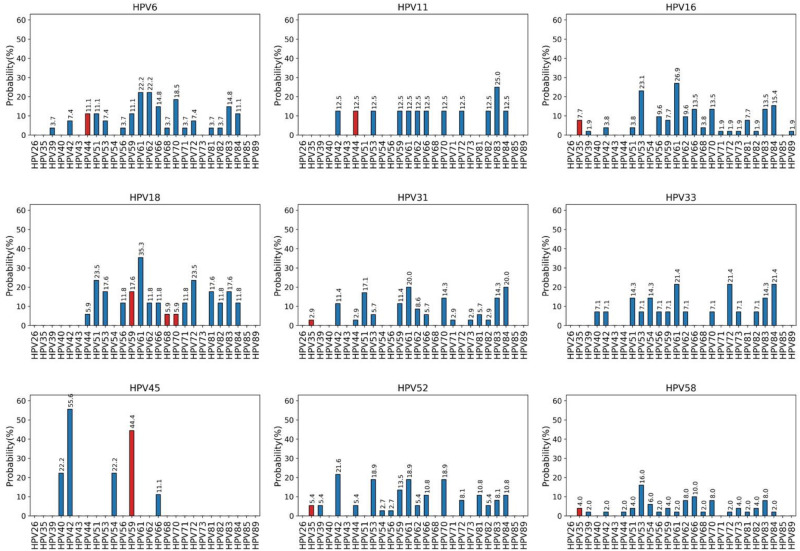
Co-occurrence probabilities. Each panel corresponds to a genotype subtype of the nonavalent vaccine indicated above the panel (reference genotype), and the columns correspond to 26 high-risk and low-risk HPV genotypes detected by the CLART2 array. In each panel, red columns represent HPV genotypes belonging to the same species of the reference genotype, and blue columns represent HPV genotypes belonging to different classes from the reference genotype.

Through an analysis of HPV MIs, we investigated the probabilities of co-occurrence of the HPV types included in the 9v (HPV6, 11, 16, 18, 31, 33, 45, 52, and 58) with the HR and LR HPV types detected by the HPV Genomica CLART2HPV-assay in cervical samples of HIV-positive women. Each of the panels in Figure 3 corresponds to a genotype subtype of the 9v HPV types indicated above the panel (reference genotype) and the further 26 HR and LR HPV types. Each panel depicts the probability *P* (*y*-axis) that a specific HR or LR HPV genotype (each column in each panel, *x*-axis) co-exists with the reference genotype. In each panel, the red columns represent HPV genotypes that belong to the same species as the reference genotype, and the blue columns represent HPV genotypes belonging to different classes from the reference genotype. *P* represents the probability that two different HPV types can occur together. Thus, a null probability between genotype A given B means that the presence of A rules out the possibility to have B. In this sense, we say that the null probability corresponds to perfect cross-protection, or even that the probability of co-occurrence is the minima.

For instance, the genotype subtype HPV6 does not occur with HPV26, 35, 40, 43, 54, 73, 85, and 89; whereas the α9 HR types (HPV16, 31, 33, 52, and 58) show similar probabilities of co-occurrence with the other α9 HPV (HPV35), or with other HPV genotypes that belong to different species. For instance, the probability of HPV35 in the presence of HPV16 is 0.077 which is the same as the probability for HPV59. The same observation holds for α7 species (HPV18, 45) that have the same probability of co-occurrence with α7 (HPV59, 68, 70) or with other α-species [i.e., C(HPV18 | HPV59) = C(HPV18 | HPV81) = 1–0.176 = 0.8].

The analysis of the results here allows us to conclude that infection by each of the 9v HPV types is associated with the presence of a specific set of HPV genotypes. In particular, these data suggest that genotype HPV6 might protect from HPV26, 35, 40, 43, 54, 73, and 85 infections; HPV11 from HPV26, 35, 39, 40, 43, 51, 54, 56, 68, 71, 73, 81, 85, and 89; HPV16 from HPV26, 40, 43, 44, 54, and 85; HPV18 from HPV26, 35, 39, 40, 42, 43, 54, 73, 85, and 89; HPV31 from HPV26, 39, 40, 43, 54, 56, 68, 72, 85, and 89; HPV33 from HPV26, 35, 39, 43, 44, 66, 68, 71, 81, 85, and 89; HPV45 from HPV26, 35, 39, 43, 44, 51, 53, 56, 61, 62, 68, 70, 71, 72, 73, 81, 82, 83, 84, 85, and 89; HPV52 from HPV26, 40, 43, 51, 68, 71, 73, 85, and 8, and HPV58 from HPV26, 40, 43, 71, and 85. The evidence that infection by at least one of the 9v HPV types is never associated with the presence of HPV26, 35, 40, 43, 54, and 73 can lead to speculate that the 9v HPV types might completely protect from co-infections by the six HPV genotypes.

**FIGURE 4:**
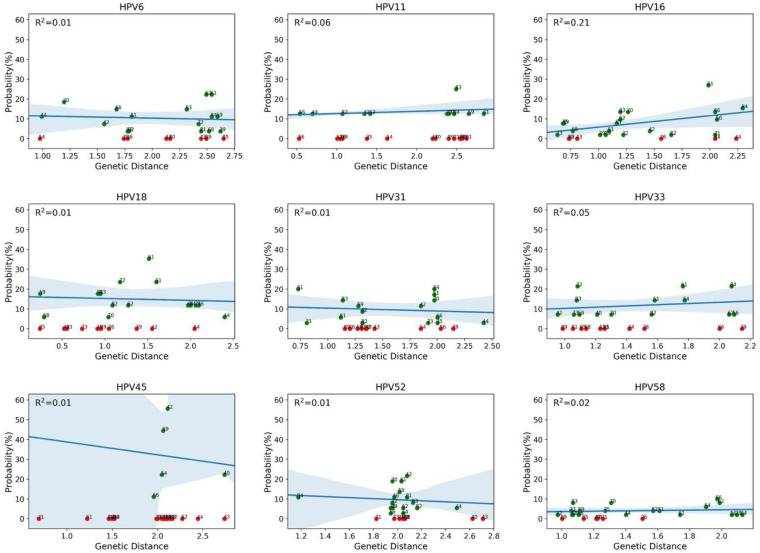
Correlations between co-occurrence probabilities and the genotype genetic distances. Each panel corresponds to a genotype subtype of the nonavalent vaccine against HPV (9v), as indicated above each panel (reference genotype). Each panel shows the scatter plot between the co-occurrence probability (*y*-axis) and the genetic distance (*x*-axis), where the dots represent the 26 high and low risk HPV genotype subtypes. The genetic distance was calculated using the Poisson method, which includes insertion and deletion. The blue line represents the least squares linear regression line, with the relative confidence intervals at 95%, and *R*^2^ represents the linear regression model fitting index.

**FIGURE 5:**
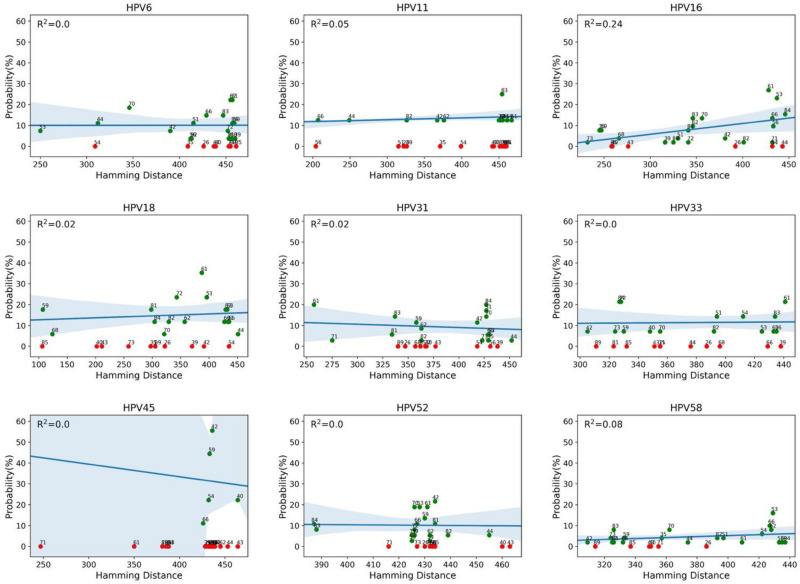
Correlation between co-occurrence probabilities and the genotype Hamming distances. Each panel corresponds to a genotype of the nonavalent vaccine against HPV (9v), as indicated above the panel (reference genotype). Each panel shows the scatter plot between the co-occurrence probability (*y*-axis) and the genotype Hamming distance (*x*-axis), where the dots represent the 26 high and low risk HPV genotypes. The blue line represents the least squares linear regression line with the relative confidence intervals at 95%, and the *R*^2^ represents the linear regression model fitting index.

**FIGURE 6:**
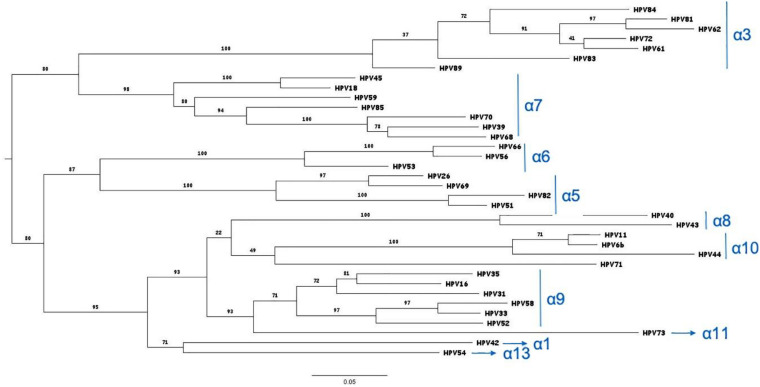
Phylogenetic tree of the HPV genotypes detected by the HPV Genomica assay. The tree was constructed on the L1 protein sequences by the maximum likelihood method: http://www.phylogeny.fr/website (phylogeny web site). The branch support values are indicated (Anisimova and Gascuel, 2006).

We then evaluated whether the co-occurrence probability correlates with the genotype distance. We used the least squares regression method to quantify this correlation. In Figure 4, each panel corresponds to a single genotype subtype of the 9v. Each panel shows the scatter plot between the co-occurrence probability (*y*-axis) and the genetic distance (*x*-axis), where the dots represent the 26 high and low risk HPV genotype subtypes. The co-occurrence probability is the same as for Figure 3, while the genetic distances were calculated using the Poisson method, which includes insertion and deletion. The blue line represents the least squares linear regression line with relative confidence intervals at 95%, the Pearson correlation and the *R*^2^ represents the linear regression model fitting index used to quantify how well the co-occurrence probability correlates with the genetic distance. In each panel, the fitting model was built considering only the genotypes with a non-null probability (Figure 4, green dots) of simultaneous occurrence with the reference genotype. Red dots correspond to genotypes with null probability of co-occurrence with the reference genotype. Figure 5 shows the same analysis in which the Hamming distances were used to quantify the distances between different genotypes. There were no linear dependencies between co-occurrence and genetic distance (Figures 4–6).

### L1 and L2 HPV81 Sequence Analysis

**FIGURE 7:**
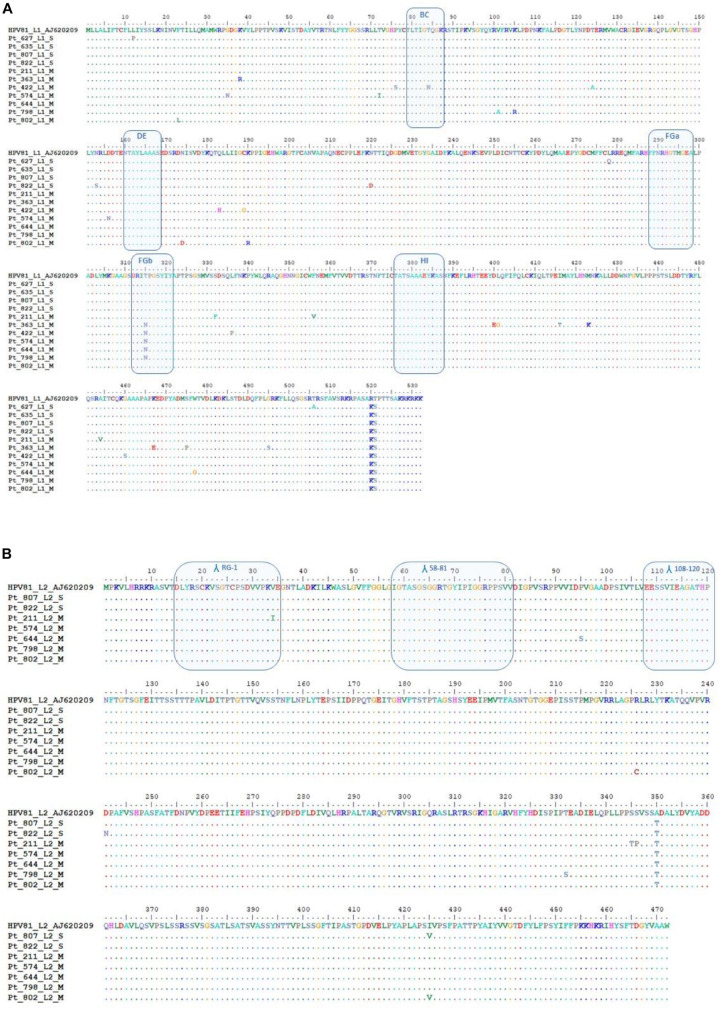
HPV81 L1 and L2 sequence analysis. **(A)** Alignment of the deduced amino-acid sequences of HPV81 L1 from four patients with HPV single infection (SI) and seven patients with HPV multiple infection (MI). The BC, DE, FGa FGb, and HI immunogenic regions that are shown on the surface of the protein are indicated in boxes. **(B)** Alignment of the deduced amino-acid sequences of HPV81 L2 from five patients with HPV MI and two patients with HPV SI. The patients from whom the sequences originated are indicated by their identification codes. The sequences were aligned with HPV81 prototype AJ620209. Dashes indicate amino-acid sequence identity with respect to the reference sequence. Λ, antigenic site.

A total of four HPV81 L1 SI (Pt627, Pt635, Pt807, and Pt822) and two HPV81 MI (Pt798, Pt802) were successfully identified (Figure 7). Five HPV81 L1 sequences were added to the analysis, from previously described MI (Pt211, Pt363, Pt422, Pt574, and Pt644; Minosse et al., 2010). The DNA sequence analysis showed 51 single nucleotide substitutions, of which 35 represented non-synonymous changes (Figure 7A; Supplementary Figure 1). The G7332A (R520K) and A7334T (T521S) changes were observed in all of the strains, and thus they can be considered as polymorphisms. Variations of the L1 surface-exposed region, which is considered to be involved in immune recognition, were analyzed (Christensen et al., 2001; Bishop et al., 2007). Among HPV81 MI, one of the seven patients (Pt422) showed an amino-acid substitution within the BC loop (N75Q), while five of the seven patients showed an amino-acid substitution in the FGb loop (T315N); no mutations were observed in HPV81 SI. All of the other amino-acid variations were randomly distributed along the L1 sequence, and here was no evidence of a premature stop codon. The β-turn prediction showed significant variability compared to the prototype only in MI (Supplementary Table 1). All of the nucleotide changes and amino-acid mutations are described in Supplementary Figure 1. Proline and glycine amino-acid changes, which influence the β-turn stability (Trevino et al., 2007), were detected only in MI (Figure 7A).

The L2 gene was successfully amplified and sequenced in two SI (Pt807, Pt822) and in five MI (Pt211, Pt574, Pt644, Pt798, and Pt802) samples. Eleven nucleotide changes were non-synonymous mutations (Supplementary Figure 1), and they resulted in nine amino-acid substitutions of the 472-amino-acids-long L2 protein (genetic variability, 1.9%; Figure 7B). All of the mutations were located outside the antigenic sites. The substitution of A350T was observed only in MI, whereas S346P was found only in two HPV81 strains of SI. For the seven HPV variants we sequenced, we observed no evidence of L2 variants that can escape immune recognition after treatment with 9v (Roden et al., 2000; Gambhira et al., 2007). The RG-1 epitope, which encompasses residues 15–34 in L2 HPV81, showed a high degree of conservation, and only Pt211 showed the I34V substitution. The furin cleavage sites (amino acids 9–12) and DNA binding motif (amino acids 1–12) were identical to the prototype in all of the analyzed specimens. No β-turn variability was seen in MI or SI L2 protein (Supplementary Table 1). Only Pt807 with a I425V substitution at the COOH carboxyterminal had a weak increase in β-turn coefficient (Supplementary Table 1).

## Discussion

HIV-positive women have a considerable risk of developing ICC, and HSIL is considered an AIDS-defining case (Maiman, 1994). The incidence of intraepithelial lesions is 8.3 per 100 HIV-positive women per year, compared to 1.8 for HIV-negative women (*p* < 0.001) (Ellerbrock et al., 2000). Increased rates of perianal, vaginal, and vulvar squamous dysplasia are also observed for HIV-positive women (Jamieson et al., 2006). The persistence of HPV infections that are linked to impairment of the immune system represents the main risk for ICC development. In HIV-positive women, ICC arises 10–15 years sooner in comparison to HIV-negative women, and it has a worse prognosis. HAART therapy and the increase in CD4 T cell counts do not appear to influence the course of the malignancy (Joseph and Chiramana, 2004; Pantanowitz et al., 2010). The 9v has been shown to be effective to prevent infections of the genotypes included in the vaccine, but it is still debated whether it provides protection against other genotypes. This is a remarkable limitation, as the persistent HPV infections observed in HIV-positive women are caused by a broad diversity of both HR and LR types (Joseph and Chiramana, 2004; De Vuyst et al., 2008; McClymont et al., 2020). These data are confirmed also by the present study: 49% of infections were represented by non9v HPV types (Figure 1). Among these, 15 HSIL were observed, where nine were SI and six were MI. Interestingly, four SI harbored HPV61, 62, and 83, which are considered to be LR types. Twenty-one LSIL specimens were SI positive for LR risk HPV42, 54, 55, 61, 62, 72, 81, 84, and 5 MI exhibited LR HPV42, 54, 61, 62, 71, and 81 (Figure 2).

Recently, Bogaards et al. re-evaluated the effectiveness of the adjuvanted bivalent HPV vaccine (2vHPV) against type-specific HPV positivity. Here, they demonstrated that cross-protection is maintained up to 8 years post-vaccination, and that the level of protection correlates with genomic distance to HPV16 or HPV18. Indeed they showed that 2vHPV can partially cross-protect against HPV types belonging to HPV α-7 (including HPV18) and α-9 (including HPV16), but cross-protection was unlikely for HPV51, 53, and 56, and for HR types belonging to the HPV α-5 or α-10 species; Bogaards et al., 2019). At variance with this, our data obtained from a real-life setting of HPV MI demonstrated that this correlation does not exist. For instance, HPV16 had a similar probability to co-exist with HPV53 (α-6) and with HPV61 (α-3). A similar probability of co-infection was observed for HPV35 (α-9) and HPV81 (α-3). HPV33 showed a comparable probability to co-exist with HPV56 (α-6) and HPV59 (α-7), irrespective of the genetic distance (Figures 3–6). HPV26, 35, 40, 43, 54, 73, and 85 are the genotypes that are completely protected by at least one of the 9v types.

A bias of this analysis might relate to the different prevalence of HPV types in different geographical areas, and thus we might have included fewer cases of an HPV type because it is not very common in Europe (e.g., HPV26, 40, 43, and 89). However, we observed that HPV types that belong to different α-species and that have similar prevalence in Italy co-existed with 9v HPV types with similar frequencies.HPV52 (α-9) infection occurred with HPV59 (α-7) and HPV81 (α-3), which had the same prevalence in a population of women (Annunziata et al., 2018). These findings suggest that the cross-protection between 9v types and other genotypes is weak or ineffective.

The absence of cross-protection against HPV types that belong to both the same and different α-species was also seen for HPV16 and HPV18 in vaccinated women from Costa Rica (Skinner et al., 2016), where HPV31 (α-9) and HPV51 (α-6) infections appeared post-vaccination. Moreover, considering that sublineage variants show significant differences in neutralization sensitivity (Godi et al., 2015; Godi et al., 2019), we examined HPV81 strains, to investigate whether different *L1* antigenic variants were present in SI and MI, and whether they might influence their cross-protection properties. Overall, the L1 protein harbored a greater number of variable amino acids than the L2 protein (35 amino-acid vs 9 amino-acid changes, respectively). For L1, six amino-acid mutations affected two antigenic sites: the BC loop (T84N, Pt422) and the FGb loop (T315N, Pt363, Pt422, Pt574, Pt644, and Pt798; Figure 7, Supplementary Table 1). In contrast, no amino-acid mutations occurred for L2 antigenic sites. In our analysis of the HPV81 L1 and L2 β-turns, variations were observed only in the L1 MI infection samples (Supplementary Table 1), which suggests modifications of interactions with antibodies and cross-reactivity between different HPV types. Indeed, several studies have demonstrated the involvement of β-turns in molecular recognition. In the immunoglobulins, the β-turns are present in the hypervariable region of CDR (Vdomain complementary determining regions), and they influence the affinity with the target antigen (Abergel et al., 1994). Single variations to β-turns impact on the conformation of proteins. Proline and glycine amino acid changes contribute to the conformational stability of β-turns (Trevino et al., 2007), and these variations were observed in L1 and MI samples. These findings suggest that the genetic differences in different settings might affect the cross-neutralization sensitivity, although this assertion should be supported by further investigations extending the analysis to more variants and to other HPV low risk types, both for SI and MI. Cross-neutralization assays should demonstrate whether lower sensitivity to cross neutralizing antibodies can be related to these variants, and a follow-up study should be conducted to determine whether lower neutralization activity has clinical relevance.

As previously indicated, the data in the literature have shown limited efficacies of the HPV vaccines against non-vaccine HPV types (Innes et al., 2018; McClymont et al., 2020). This leads to consideration of the need for anti-HPV vaccine implementation. The RG-1-L2 epitope might be a candidate to reach this goal. To date, an L2-based vaccine has never been introduced into clinical practice because although it induces neutralizing antibodies against a broad spectrum of genotypes, the antibody titer is lower than that induced by the L1 protein (Chandrachud et al., 1995; Gambhira et al., 2007). RG1-L2 (amino acids, 17-36) of HPV16 was inserted into the L1 DE loop. The chimeric VLPs induced cross neutralizing antibodies that were effective against most of the HR genotypes, or potentially carcinogenic types (HPV16, 18, 45, 31, 33, 35, 52, 58, 39, 51, 59, 68, 73, 26, 53, and 66), and LR HPV6, 43, 44 (Schellenbacher et al., 2009, 2013). In another study, amino acids 17-36 of HPV33 L2 were positioned into the HPV18 L1 DE loop, and amino acids 56-75 of the HPV58 L2 protein were inserted into the L1-COOH terminus. These VLPs induced persistent immune responses against the HPV6, 11, 16, 31, 35, 39, 45, 58, and 59 genotypes in both mice and rabbits (Boxus et al., 2016). Sequence conservation of L2 proteins allows a broadly protective response to be elicited using a few epitopes (Roden et al., 2000). The HPV81 analysis in SI and MI settings confirmed the high conservation of the L2 protein, showing no variability in antigenic sites, strengthening the usefulness of the L2 protein in HPV vaccine implementation.

## Conclusion

In summary, this study on cervical specimens from HIV-positive women showed a high prevalence of LSIL and HSIL that are related to non9v HPV types, and several of these are considered LR types. The analysis of co-infectivity starting from the real-life setting suggests that 9v types do not protect against other genotypes, and thus a vaccination campaign cannot fully prevent HPV-related intra-epithelial lesions. Therefore, these data confirm the importance of a sustainable and effective screening program, which should be implemented through HPV DNA testing that does not include only HR types.

## Data Availability Statement

The sequences of the L1 and L2 genes were submitted to the NCBI GenBank database, and were assigned accession numbers (MT547553–MT547561).

## Ethics Statement

The studies involving human participants were reviewed and approved by Institutional Ethical Committee L Spallanzani code42/13, 2013. The patients/participants provided their written informed consent to participate in this study.

## Author Contributions

ARG, PDP, and PP conceived and designed the study. CS and DL collected the samples and carried out sample HPV typing. FDN processed the cythological samples. CS, VG, CM, DL, and FDN analyzed the data. ARG and VG writing original draft. ARG, PDP, PP, VG, and MC edited the manuscript. All authors read and agreed to the final version of manuscript.

## Conflict of Interest

The authors declare that the research was conducted in the absence of any commercial or financial relationships that could be construed as a potential conflict of interest.
