# Supplementary material for: Human Papillomavirus Infections in Cervical Samples From HIV-Positive Women: Evaluation of the Presence of the Nonavalent HPV Genotypes and Genetic Diversity
Source: Front Microbiol. 2020 Nov 25;11:603657. doi: 10.3389/fmicb.2020.603657 (PMC7723855; doi:10.3389/fmicb.2020.603657)
Supplement: Supplementary file 1 [file Data_Sheet_1.PDF]

# *Supplementary Material*

**Supplementary Materials**

**Method of Least Squares_supplementary data 1**

The method of least squares is a procedure to determine the line that best fits the data. Given data $\{(x_1, y_1), \ldots, (x_N, y_N)\}$, we can define the error associated to $y = ax + b$ by:

$$E(a,b) = \sum_{n=1}^{N} \left(y_n - (ax_n + b)\right)^2.$$

The goal is to find the values of *a* and *b* that minimize the error. In multivariable calculus this requires to find the values of (*a*, *b*) such that:

$$\frac{\partial E}{\partial a} = 0, \qquad \frac{\partial E}{\partial b} = 0$$

Differentiating *E*(*a*, *b*) yields:

$$\frac{\partial E}{\partial a} = \sum_{n=1}^{N} 2\left(y_n - (ax_n + b)\right) \cdot (-x_n)$$

$$\frac{\partial E}{\partial b} = \sum_{n=1}^{N} \left(y_n - (ax_n + b)\right)$$

Setting $\partial E/\partial a = \partial E/\partial b = 0$ (and dividing by 2) yields:

$$\sum_{n=1}^{N}\left(y_n - (ax_n + b)\right) \cdot x_n = 0$$

$$\sum_{n=1}^{N}\left(y_n - (ax_n + b)\right) = 0$$

We can rewrite these equations as:

$$\left(\sum_{n=1}^{N} x_n^2\right) a + \left(\sum_{n=1}^{N} x_n\right) b = \sum_{n=1}^{N} x_n y_n$$

$$\left(\sum_{n=1}^{N} x_n\right) a + \left(\sum_{n=1}^{N} 1\right) b = \sum_{n=1}^{N} y_n$$

Here the values of *a* and *b* that minimize the error *E*(*a*,*b*) satisfy the following matrix equation:

$$\begin{pmatrix} \sum_{n=1}^{N} x_n^2 & \sum_{n=1}^{N} x_n \\ \sum_{n=1}^{N} x_n & \sum_{n=1}^{N} 1 \end{pmatrix} \begin{pmatrix} a \\ b \end{pmatrix} = \begin{pmatrix} \sum_{n=1}^{N} x_n y_n \\ \sum_{n=1}^{N} y_n \end{pmatrix}$$

Where we can define

$$M = \begin{pmatrix} \sum_{n=1}^{N} x_n^2 & \sum_{n=1}^{N} x_n \\ \sum_{n=1}^{N} x_n & \sum_{n=1}^{N} 1 \end{pmatrix}$$

the determinant of which is:

$$\det(M) = N\sum_{n=1}^{N} x_n^2 - (N\bar{x})^2 = N^2\left(\frac{1}{N}\sum_{n=1}^{N} x_n^2 - \bar{x}^2\right) = N^2\frac{1}{N}\sum_{n=1}^{N}(x_n - \bar{x})^2$$

If $M$ is invertible (i.e., det($M$) is non-zero), the solution is:

$$\binom{a}{b} = \begin{pmatrix} \sum_{n=1}^{N} x_n^2 & \sum_{n=1}^{N} x_n \\ \sum_{n=1}^{N} x_n & \sum_{n=1}^{N} 1 \end{pmatrix}^{-1} \begin{pmatrix} \sum_{n=1}^{N} x_n y_n \\ \sum_{n=1}^{N} y_n \end{pmatrix}$$

**TABLE S1.** Data on the β-turn values.

| **Code** | **Region** | **Infection** | **β-Turn value** |
|---|---|---|---|
| Pt_211 | L1 | MI | 8.93 |
| Pt_363 | L1 | MI | 10.71 |
| Pt_422 | L1 | MI | 8.50 |
| Pt_574 | L1 | MI | 8.73 |
| Pt_644 | L1 | MI | 8.73 |
| Pt_798 | L1 | MI | 8.73 |
| Pt_802 | L1 | MI | 9.92 |
| Pt_822 | L1 | SI | 9.33 |
| Pt_627 | L1 | SI | 9.52 |

| | | | |
|---|---|---|---|
| Pt_635 | L1 | SI | 9.33 |
| Pt_807 | L1 | SI | 9.33 |
| AJ62209 | L1 | | 9.44 |
| Pt_211 | L2 | MI | 5.08 |
| Pt_574 | L2 | MI | 5.08 |
| Pt_644 | L2 | MI | 5.08 |
| Pt_798 | L2 | MI | 5.08 |
| Pt_802 | L2 | MI | 5.08 |
| Pt_822 | L2 | SI | 5.08 |
| Pt_807 | L2 | SI | 5.51 |
| AJ62209 | L2 | | 5.08 |

MI, multiple infection; SI, single infection, Prototype, AJ62209

**Panel A**

| | nucleotide position | 5808 | 5840 | 5876 | 5877 | 5886 | 5988 | 5999 | 6024 | 6028 | 6043 | 6075 | 6087 | 6143 | 6231 | 6239 | 6293 | 6319 | 6322 | 6328 | 6338 | 6342 | 6431 | 6499 | 6511 | 6514 | 6606 | 6717 |
|---|---|---|---|---|---|---|---|---|---|---|---|---|---|---|---|---|---|---|---|---|---|---|---|---|---|---|---|---|
| | **AJ620209** | T | T | G | G | A | C | C | C | A | T | T | A | A | A | G | A | A | A | A | T | A | A | G | G | G | T | C |
| **Single infections** | **Pt_627** | C | - | - | - | - | - | - | - | - | - | - | - | - | - | - | - | - | - | - | - | - | - | - | A | - | A | - |
| | **Pt_635** | - | - | - | - | - | - | - | - | - | - | - | - | - | - | - | - | - | - | - | - | - | - | - | A | - | - | - |
| | **Pt_807** | - | - | - | - | - | - | - | - | - | - | - | - | - | - | - | - | - | - | - | - | - | - | - | A | - | - | - |
| | **Pt_822** | - | - | - | - | - | - | - | - | - | - | - | - | - | G | - | - | - | - | - | - | - | G | - | A | - | - | - |
| **Multiple infections** | **Pt_211** | - | - | - | - | - | - | - | - | - | C | - | - | - | - | - | - | - | - | - | - | - | - | - | - | - | - | - |
| | **Pt_363** | - | - | - | - | G | - | - | - | - | | - | - | - | - | - | - | - | - | - | - | - | - | A | A | A | - | A |
| | **Pt_422** | - | - | - | - | - | - | T | A | - | - | - | - | G | - | - | - | C | T | - | G | - | - | - | A | - | - | A |
| | **Pt_574** | - | - | A | A | - | T | - | - | G | - | - | - | - | - | A | - | - | - | G | - | - | - | - | C | - | - | A |
| | **Pt_644** | - | - | - | - | - | - | - | - | - | - | - | - | - | - | - | - | - | - | - | - | - | - | - | T | - | - | A |
| | **Pt_798** | - | - | - | - | - | - | - | - | - | - | C | G | - | - | - | - | - | - | - | - | - | - | - | T | - | - | A |
| | **Pt_802** | - | C | - | - | - | - | - | - | - | - | - | - | - | - | - | G | - | - | - | - | G | - | - | A | - | - | - |
| | | ↓ | ↓ | ↓ | | ↓ | ↓ | ↓ | ↓ | | | ↓ | ↓ | ↓ | ↓ | ↓ | ↓ | | ↓ | | ↓ | ↓ | ↓ | | | | ↓ | ↓ |
| | **aa on ref** | L | F | G | | K | T | P | T | | | V | K | T | N | D | N | | Q | | C | K | N | | | | L | T |
| | **aa position** | 12 | 23 | 35 | | 38 | 72 | 76 | 84 | | | 101 | 105 | 124 | 153 | 156 | 174 | | 183 | | 189 | 190 | 220 | | | | 278 | 315 |
| | **aa mutation** | P | L | N | | R | I | S | N | | | A | R | A | S | N | D | | H | | G | R | D | | | | Q | N |

**Panel B**

| | nucleotide position | 6724 | 6768 | 6780 | 6793 | 6839 | 6859 | 6919 | 6950 | 6973 | 6974 | 6975 | 7020 | 7041 | 7123 | 7134 | 7147 | 7151 | 7172 | 7196 | 7202 | 7356 | 7279 | 7289 | 7297 | 7332 | 7334 |
|---|---|---|---|---|---|---|---|---|---|---|---|---|---|---|---|---|---|---|---|---|---|---|---|---|---|---|---|
| | **AJ620209** | G | C | T | T | T | A | A | C | T | T | T | T | T | A | C | A | G | A | T | T | G | T | A | T | G | A |
| **Single infections** | **Pt_627** | A | - | - | - | - | - | - | - | - | - | - | - | - | G | - | G | - | - | - | - | - | - | G | - | A | T |
| | **Pt_635** | A | - | - | - | - | - | - | - | - | - | - | - | - | - | - | G | - | - | - | - | - | - | - | - | A | T |
| | **Pt_807** | A | - | - | - | - | - | - | - | - | - | - | - | - | - | - | G | - | - | - | - | - | - | - | - | A | T |
| | **Pt_822** | A | - | - | - | - | - | - | - | - | - | - | - | - | - | - | G | - | - | - | - | - | G | - | - | A | T |
| **Multiple infections** | **Pt_211** | - | T | - | - | G | - | - | - | - | - | - | - | - | - | T | - | - | - | - | - | - | - | - | - | A | T |
| | **Pt_363** | - | - | - | - | - | - | - | T | G | G | G | C | A | - | - | - | - | G | C | - | A | - | - | - | A | T |
| | **Pt_422** | - | - | C | C | - | - | - | - | - | - | - | - | - | - | - | - | A | - | - | - | - | - | - | G | A | T |
| | **Pt_574** | - | - | - | - | - | G | - | - | - | - | - | - | - | - | - | - | - | - | - | - | - | - | - | A | A | T |
| | **Pt_644** | - | - | - | - | - | - | - | - | - | - | - | - | - | - | - | - | - | - | - | G | - | - | - | - | A | T |
| | **Pt_798** | - | - | - | - | - | - | - | - | - | - | - | - | - | - | - | - | - | - | - | - | - | - | - | - | A | T |
| | **Pt_802** | - | - | - | - | - | - | G | - | - | - | - | - | - | - | - | G | - | - | - | - | - | - | - | - | A | T |
| | | | ↓ | ↓ | | ↓ | | | | ↓ | ↓ | | ↓ | ↓ | | ↓ | | ↓ | ↓ | ↓ | ↓ | ↓ | | ↓ | | ↓ | ↓ |
| | **aa on ref** | | S | L | | F | | | | D | L | | I | M | | A | | G | K | S | W | G | | T | | R | T |
| | **aa position** | | 332 | 336 | | 356 | | | | 400 | 401 | | 416 | 423 | | 454 | | 460 | 467 | 475 | 477 | 495 | | 506 | | 520 | 521 |
| | **aa mutation** | | F | P | | V | | | | E | G | | T | K | | V | | S | E | P | G | S | | A | | K | S |

**Panel C**

| | nucleotide position | 4561 | 4744 | 4746 | 4950 | 4962 | 5133 | 5137 | 5182 | 5455 | 5490 | 5493 | 5494 | 5496 | 5497 | 5509 | 5601 | 5734 | 5781 |
|---|---|---|---|---|---|---|---|---|---|---|---|---|---|---|---|---|---|---|---|
| | **AJ620209** | G | C | G | T | A | G | C | G | A | A | T | T | C | T | G | A | A | T |
| **Single infections** | **Pt_807** | - | - | A | - | - | A | - | - | - | - | - | - | - | - | A | - | G | - |
| | **Pt_822** | - | - | A | C | - | - | - | A | - | - | - | - | - | - | A | - | - | - |
| **Multiple infections** | **Pt_211** | A | - | A | - | C | - | - | - | - | T | C | A | A | C | A | - | - | - |
| | **Pt_574** | - | - | A | - | - | - | - | - | - | - | - | - | - | - | A | - | - | - |
| | **Pt_644** | - | T | A | - | - | - | - | - | - | - | - | - | - | - | A | C | - | - |
| | **Pt_798** | - | - | A | - | - | - | - | - | T | - | - | - | - | - | A | - | - | - |
| | **Pt_802** | - | - | A | - | - | - | T | - | - | - | - | - | - | - | A | - | G | C |
| | | ↓ | ↓ | | | | | ↓ | ↓ | ↓ | | | ↓ | | ↓ | ↓ | | ↓ | |
| | **aa on ref** | V | P | | | | | R | D | T | | | S | | S | A | | I | |
| | **aa position** | 34 | 95 | | | | | 226 | 241 | 332 | | | 345 | | 346 | 350 | | 425 | |
| | **aa mutation** | I | S | | | | | C | N | S | | | T | | P | T | | V | |

**FIGURE S1**. **Nucleotide sequence variations among the HPV81 strains in the L1 (A, B) and L2 (C) open reading frame**. Nucleotide positions at which variations were observed are written vertically at the top. The numbering of the nucleotides refers to the nucleotide sequence of HPV81 prototype AJ620209 Variable. The nucleotide positions within the same codon are underlined and amino acid changes resulting from nonsynonymous mutations are indicated at the bottom.
